# Supplementary material for: Shoot Regeneration Is Not a Single Cell Event
Source: Plants (Basel). 2020 Dec 29;10(1):58. doi: 10.3390/plants10010058 (PMC7823732; doi:10.3390/plants10010058)
Supplement: Supplementary file 1 [file plants-10-00058-s001.pdf]

Supplemental data

Shoot regeneration is not a single cell event

Patharajan Subban, Yaarit Kutsher, Dalia Evenor, Eduard Belausov, Hanita Zemach, Adi Faigenboim, Samuel Bocobza, Mike Timko, Moshe Reuveni.

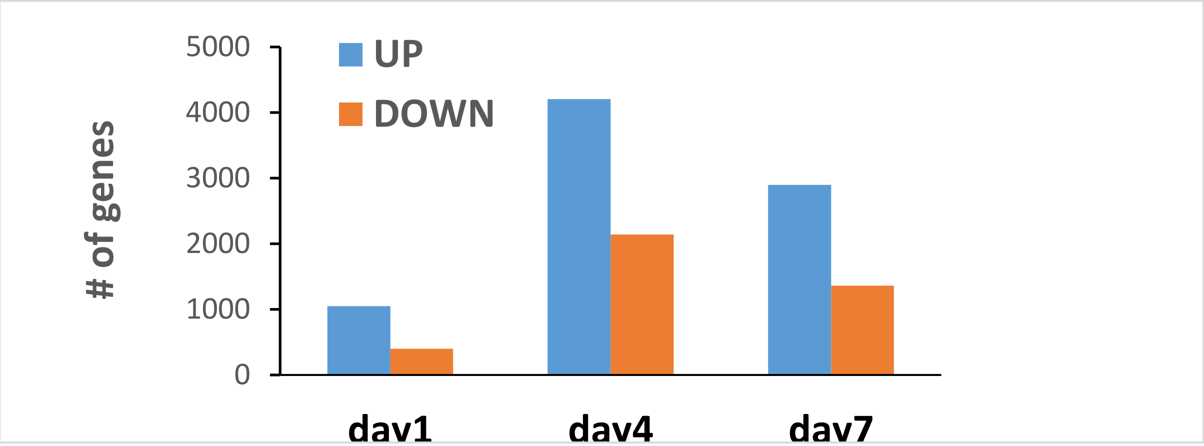

Figure S1: Number of genes that were up or down-regulated during the induction period on Reg medium.

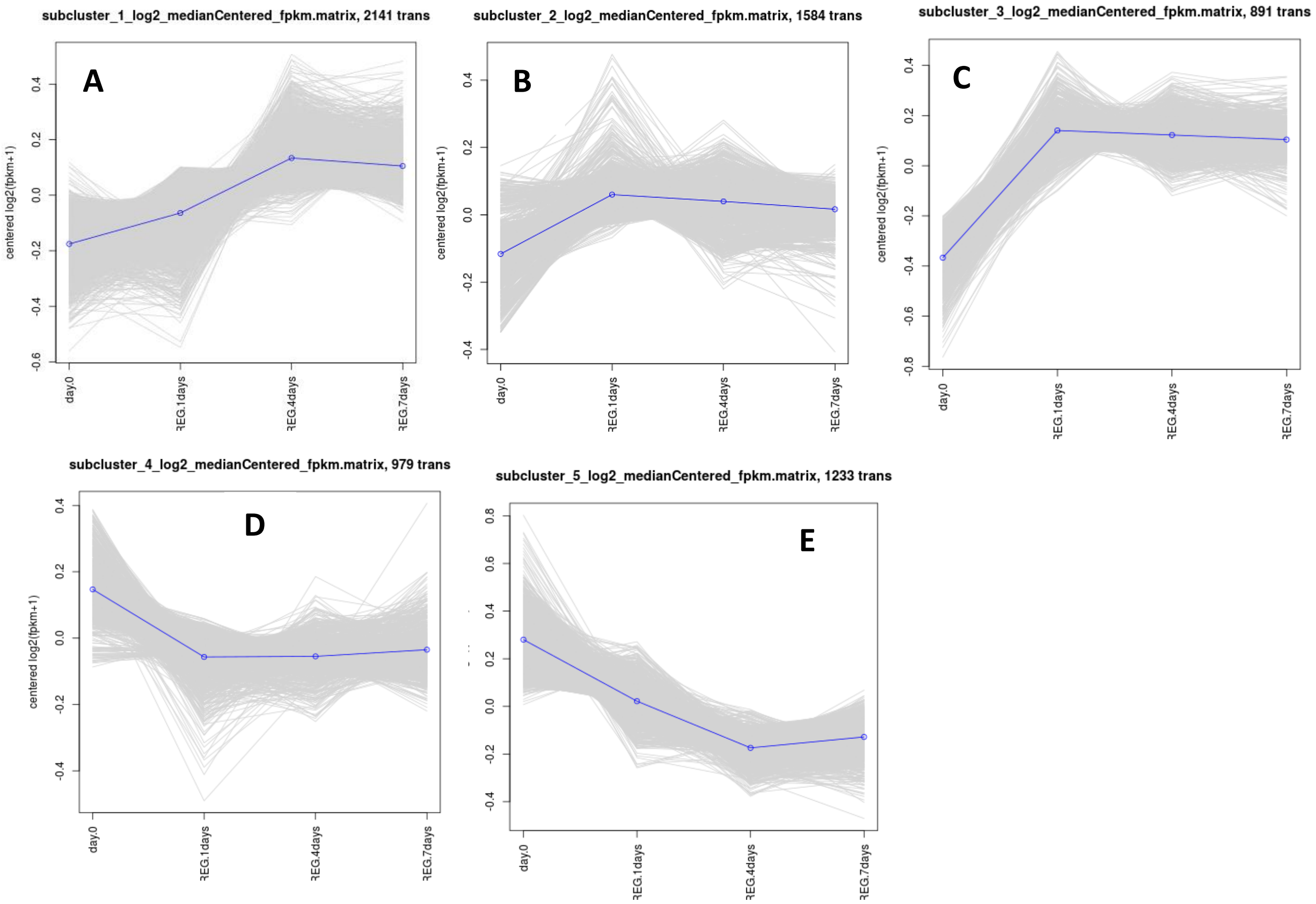

Fig. S2: Transcript changes during shoot induction period on Reg medium show five distinct sub cluster. **A**: sub cluster of genes that upregulate mostly on day 4; **B**: sub cluster of genes that upregulate moderately on day 1; **C**: sub cluster of genes that upregulate sharply on day 1; **D**: sub cluster of genes that downregulate on day 1; **E**: sub cluster of genes that downregulate on day 4.

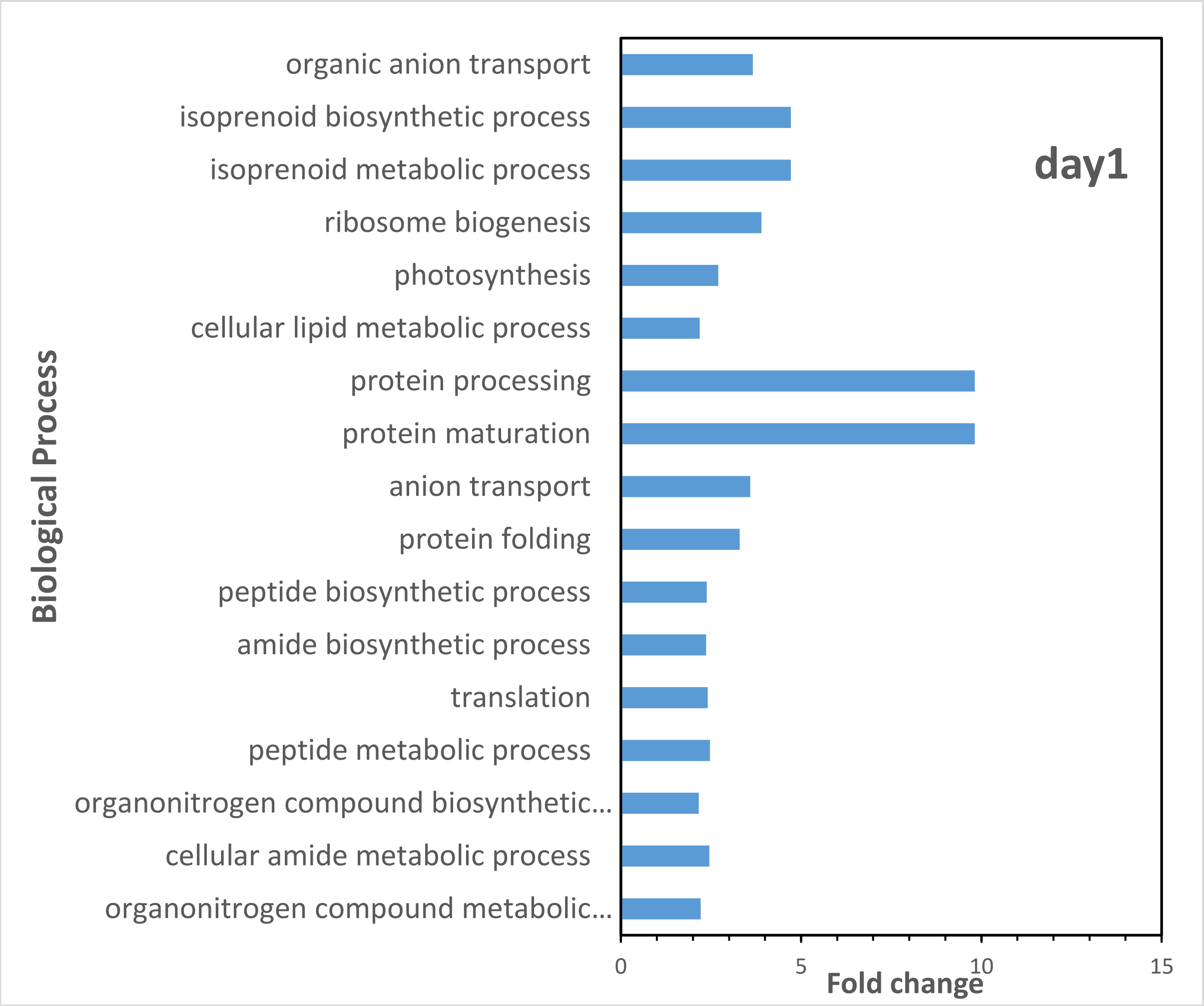

Figure S3. transcript analyses of biological processes that upregulate at day 1 compared to day 0 of the induction of shoot regeneration.

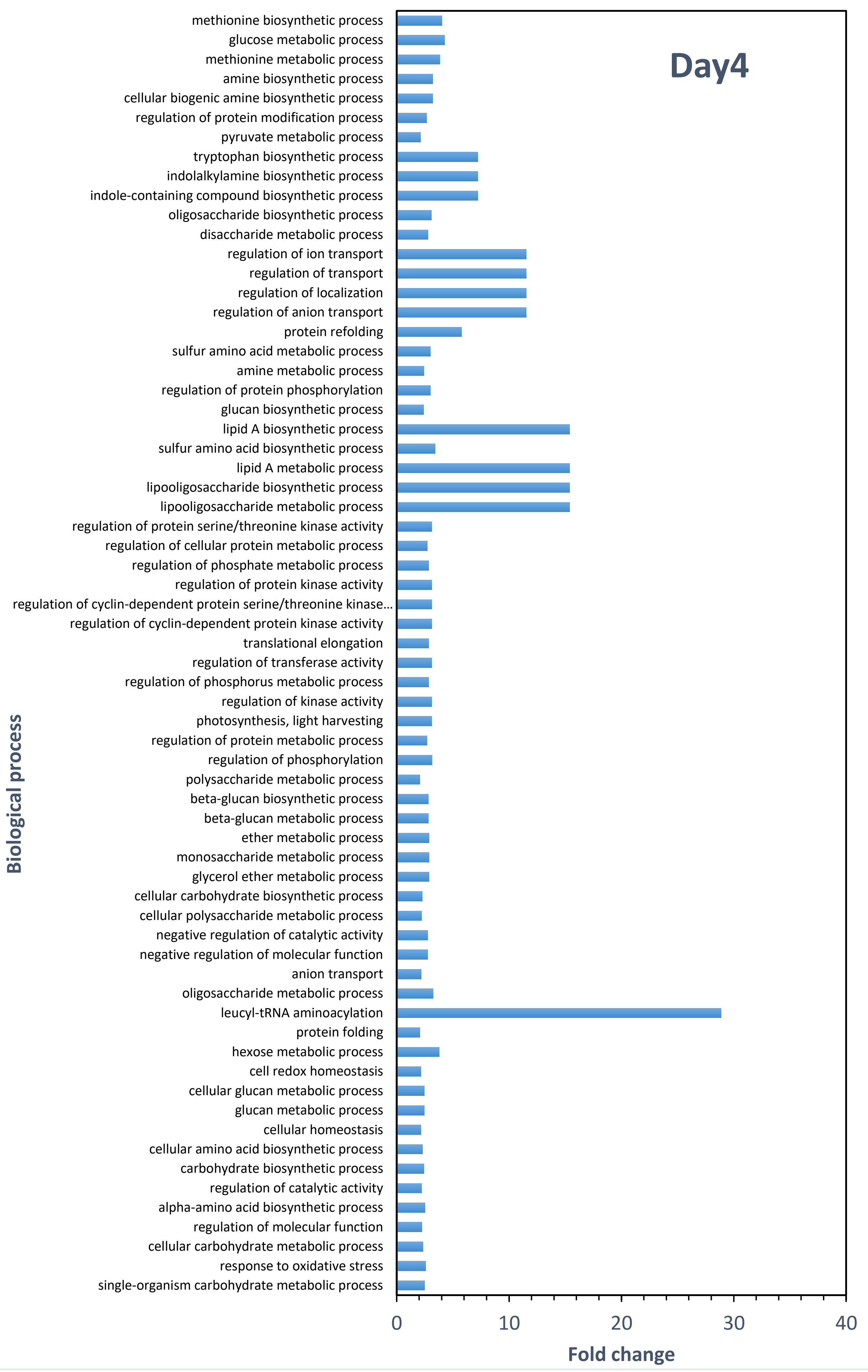
Day4
methionine biosynthetic process
glucose metabolic process
methionine metabolic process
amine biosynthetic process
cellular biogenic amine biosynthetic process
regulation of protein modification process
pyruvate metabolic process
tryptophan biosynthetic process
indolalkylamine biosynthetic process
indole-containing compound biosynthetic process
oligosaccharide biosynthetic process
disaccharide metabolic process
regulation of ion transport
regulation of transport
regulation of localization
regulation of anion transport
protein refolding
sulfur amino acid metabolic process
amine metabolic process
regulation of protein phosphorylation
glucan biosynthetic process
lipid A biosynthetic process
sulfur amino acid biosynthetic process
lipid A metabolic process
lipooligosaccharide biosynthetic process
lipooligosaccharide metabolic process
regulation of protein serine/threonine kinase activity
regulation of cellular protein metabolic process
regulation of phosphate metabolic process
regulation of protein kinase activity
regulation of cyclin-dependent protein serine/threonine kinase…
regulation of cyclin-dependent protein kinase activity
translational elongation
regulation of transferase activity
regulation of phosphorus metabolic process
regulation of kinase activity
photosynthesis, light harvesting
regulation of protein metabolic process
regulation of phosphorylation
polysaccharide metabolic process
beta-glucan biosynthetic process
beta-glucan metabolic process
ether metabolic process
monosaccharide metabolic process
glycerol ether metabolic process
cellular carbohydrate biosynthetic process
cellular polysaccharide metabolic process
negative regulation of catalytic activity
negative regulation of molecular function
anion transport
oligosaccharide metabolic process
leucyl-tRNA aminoacylation
protein folding
hexose metabolic process
cell redox homeostasis
cellular glucan metabolic process
glucan metabolic process
cellular homeostasis
cellular amino acid biosynthetic process
carbohydrate biosynthetic process
regulation of catalytic activity
alpha-amino acid biosynthetic process
regulation of molecular function
cellular carbohydrate metabolic process
response to oxidative stress
single-organism carbohydrate metabolic process
Biological process
0
10
20
30
40
Fold change

Figure S4. transcript analyses of biological processes that upregulate at day 4 compared to day 0 of the induction of shoot regeneration.

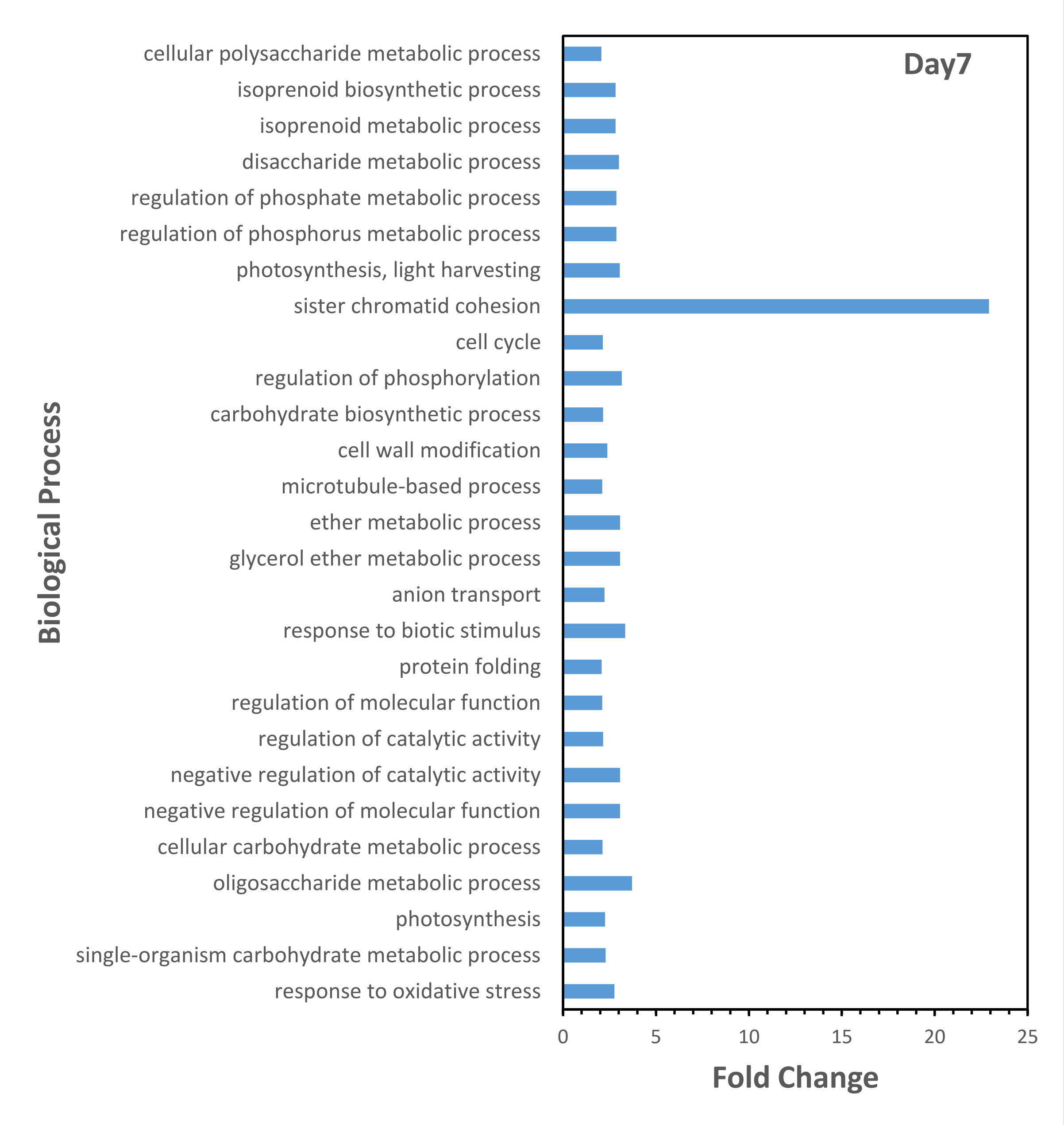

Figure S5. Transcript analyses of biological processes that upregulate at day 7 compared to day 0 of the induction of shoot regeneration.

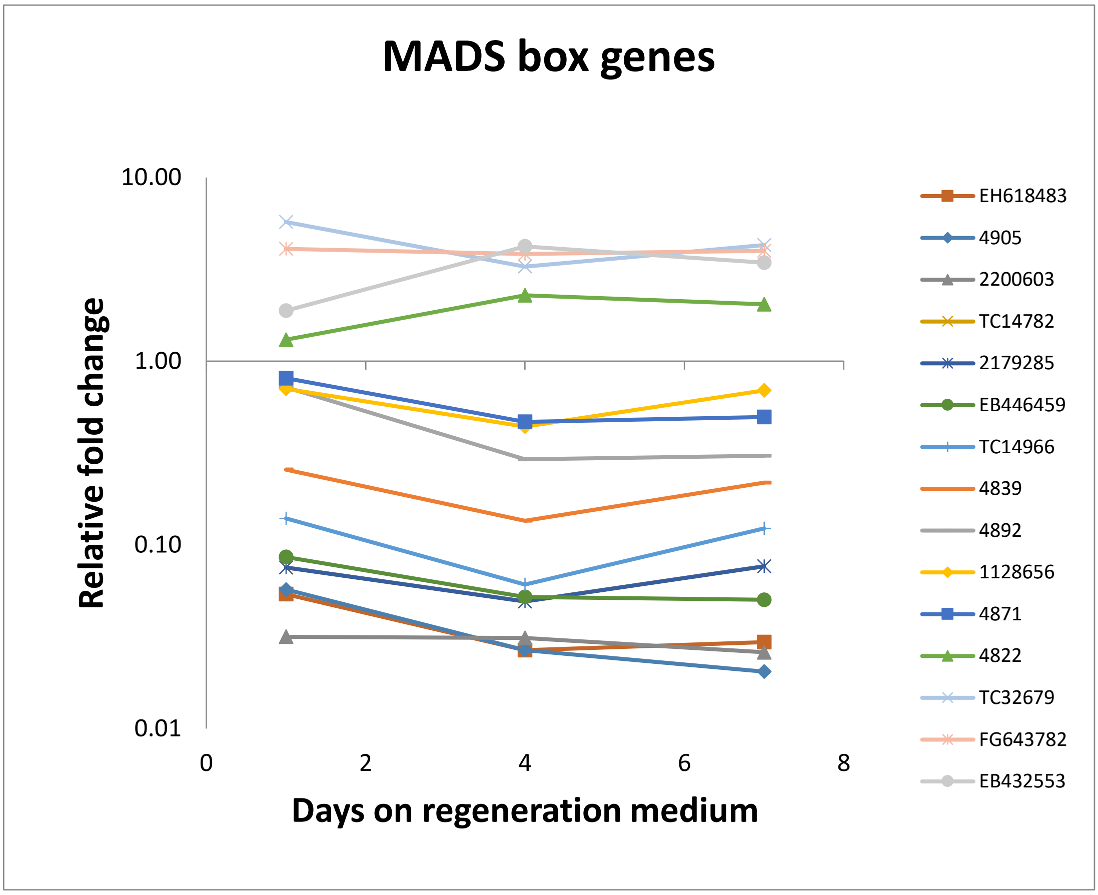

Figure S6. Transcript analyses of MADS box gene family during the 7 c days ompared to day 0 of the induction of shoot regeneration.

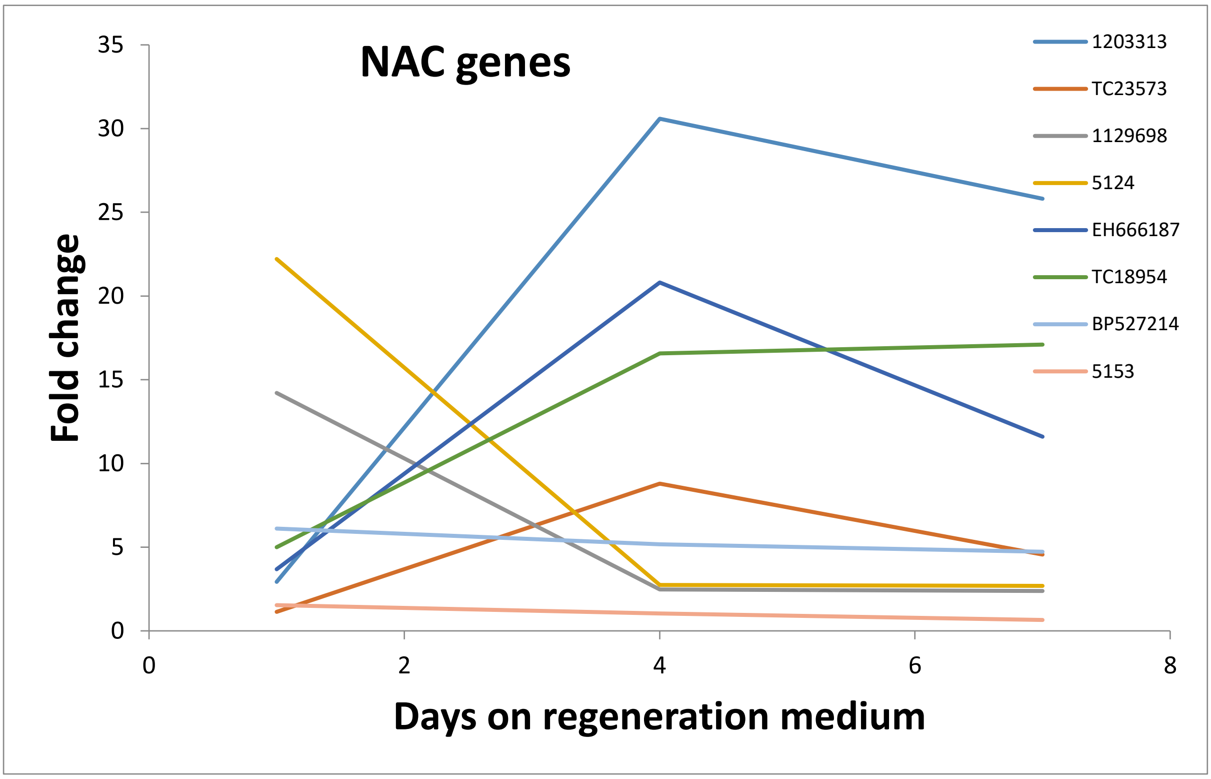

Figure S7. Transcript analyses of NAC gene family during the 7 c days ompared to day 0 of the induction of shoot regeneration.

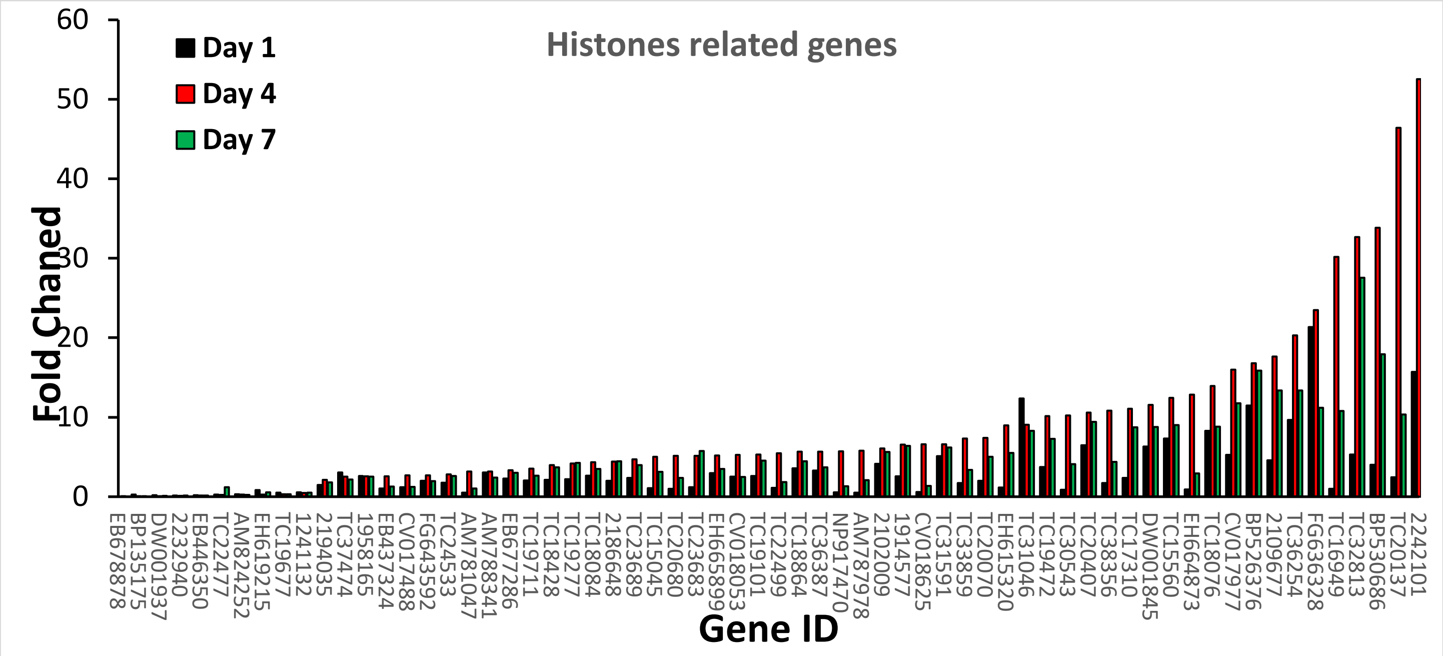

Figure S8. Transcript analyses of histone related genes during the 7 c days ompared to day 0 of the induction of shoot regeneration.

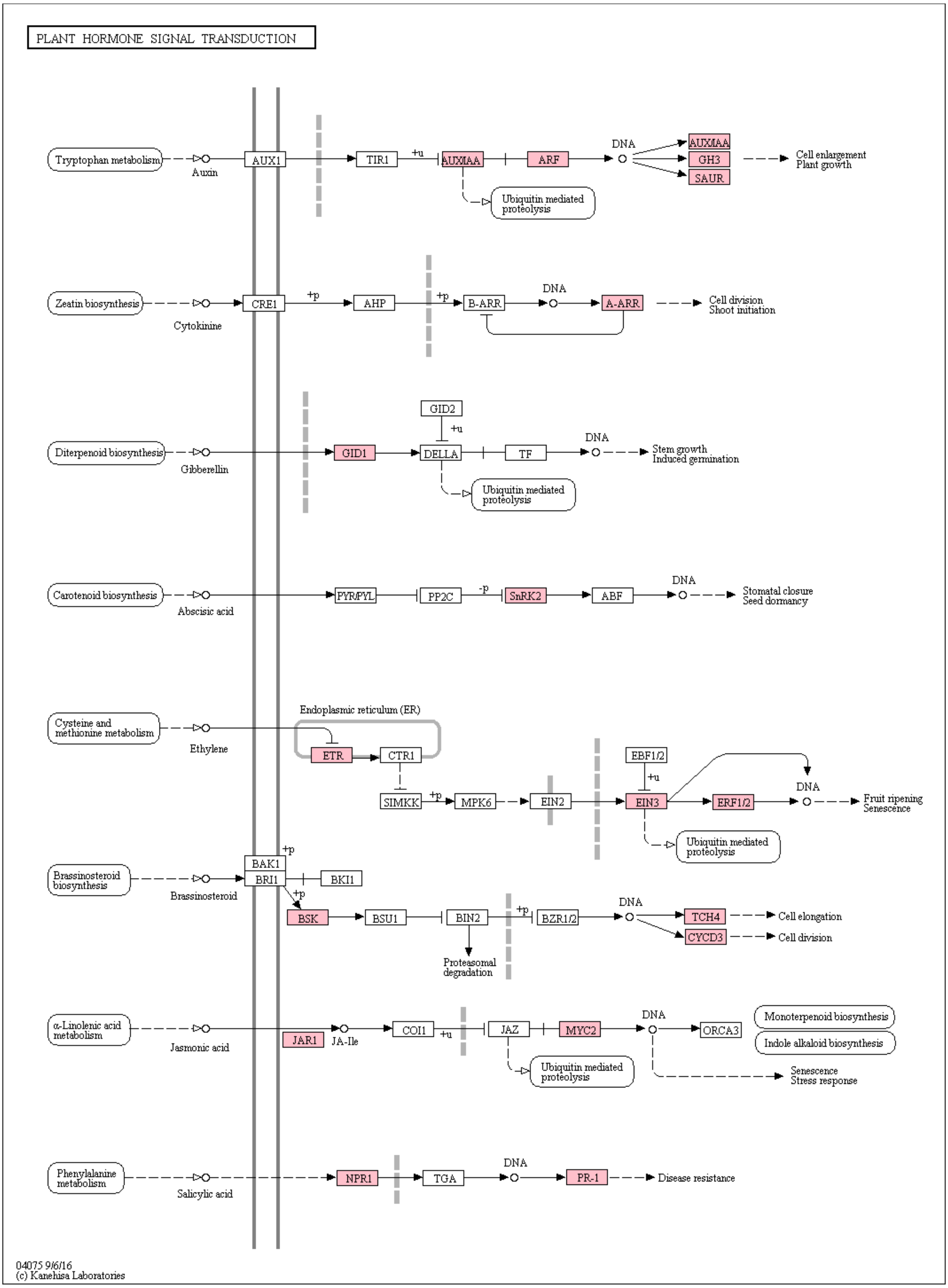

Figure S9. Transcript analyses of phytohormones signal transduction phatways at the 1 day ompared to day 0 of the induction of shoot regeneration. Genes that are boxed in red show change.
